# The Psychosocial Impact of Lower Limb Amputation on Patients and Caregivers

**DOI:** 10.7759/cureus.31248

**Published:** 2022-11-08

**Authors:** Mohammed Alessa, Hassan A Alkhalaf, Salsabeel S Alwabari, Noor J Alwabari, Hussain Alkhalaf, Zahraa Alwayel, Fatimah Almoaibed

**Affiliations:** 1 General Surgery, King Faisal University, Al-Ahsa, SAU; 2 Surgery, King Faisal University, Al-Ahsa, SAU; 3 Medical Student, King Faisal University, Al-Ahsa, SAU; 4 Nursing, Imam Abdulrahman Bin Faisal University, Khobar, SAU; 5 Dermatology, King Faisal University, Al-Ahsa, SAU; 6 Neurology, King Faisal University, Al-Ahsa, SAU

**Keywords:** prosthesis, lower limb amputation, burden, quality of life, depression

## Abstract

Introduction

The impact of amputation on patients' social and psychological well-being has been demonstrated. However, the experiences and requirements of amputees during the adjustment phase vary between amputees.

Methods

This study aimed to assess how amputation affects psychosocial life and the quality of life (QOL) in the amputees, the psychosocial processes involved in adjusting to amputation and a prosthesis, and the burden of amputees on caregivers in Saudi Arabia. A cross-sectional study was placed from November 2021 to February 2022, and it included all amputees and caregivers available at the time of the study.

Result

A total of 239 amputees and 219 caregivers were included in the study. The average level of the physical component score (PCS) was 63.5% ± 14.6% and 57.3% ± 12.9% for the mental component score (MCS). There is a significant positive correlation between psychological adjustment total and satisfaction with prosthesis with PCS and MCS dimension of QOL. Considering the QOL, PCS mean score was significantly higher among prosthetics users than among non-users (68.2 ± 15.5 vs. 59.9 ± 12.8, respectively; P=.001). Also, the MCS score was significantly higher among prosthetics users than among non-users (59.5 ± 12.4 vs. 55.5 ± 13.0, respectively; P=.001). A total of 15.1% of caregivers experienced a high burden, while 23.3% had a mild to moderate burden, but 61.6% had no or little burden.

Conclusion

Our finding shows there are correlations between psychological adjustment total and satisfaction with prosthesis with PCS and MCS dimension of QOL. The findings emphasize the importance of psychological and social support to be considered in caregivers’ health assessments. Also, the physical health of the caregivers should be fundamental in their lives as it minifies the caregiver burden. Further studies should be considered with a larger sample of amputees and longitudinal studies to evaluate the adaptation changes over time, caregiving burden, and family functioning.

## Introduction

Amputation results in permanent disability and a significant alteration in the individual's life and performance. Amputations of the upper and lower extremities are connected with psychological problems and different levels of physical impairment [1]. Lower limb amputation (LLA) affects an individual's social and psychological, and well-being in addition to causing lasting physical changes. As a result, it can affect various elements that influence a person's Health Related Quality of Life (HRQOL) [2,3]. LLA accounted for 73.5% of all limb losses, with traumatic injuries and vascular disease accounting for most cases [4]. LLAs are mainly an unavoidable procedure in the late stages of several diseases, including peripheral arterial occlusive disease (PAOD), diabetes mellitus (DM), oncological disorders, infection, or trauma, and acts as a dramatic influence on a patient's life. Amputation incidences in various countries vary from 1.2 to 4.4 per 10,000 people [5,6]. In 2018, medical rehabilitation facilities and departments in Saudi Arabia recorded 3745 incidents of amputation [7]. Diabetes was the primary cause of more than half of these cases, while other factors involved malignancies, traffic accidents, and other accidents. Due to undisclosed reasons, two-thirds of amputees do not obtain rehabilitation assistance [8]. Vascular problems are the leading cause of LLAs in developed countries [5,9,10], whereas traumatic accidents are the leading cause of amputation in developing countries [10]. After an amputation, a person's body structure and function are altered, which affects their level of activity and involvement in society [11]. Amputation results and the long-term functioning of amputees are also influenced by environmental and personal factors [11,12]. An important factor in amputation recovery is psychosocial support, which has been shown to positively impact recovery [13]. Prostheses can have a major impact on patients' participation, movement, and psychological functioning following amputation, therefore altering their quality of life (QOL) [14-16]. QOL was shown to be improved by the absence of phantom pain, residual limb pain, and the ability to walk longer distances [17]. Depression is one of the most widely utilized indicators of psychosocial response following amputation. Some patients have stated that depression caused them to wear their prostheses less frequently in the period following their amputation [18]. Patients with long-term amputations are more likely to be depressed, linked to more activity restrictions [19], and feelings of weakness [20]. Research has repeatedly noticed that individuals with LLAs have higher levels of anxiety and depression than the general population [13,21]. LLAs results in emotional, psychological, physical, and physical problems, needing caregiver help with rehabilitation and general health care [22-24]. Amputation's effects on patients' families may be better understood by the medical community if caregivers' enormous burdens are highlighted and their frequently unmet demands are brought to light [25]. The impact of amputation on a patient's family can be better understood by highlighting the heavy burden carried by caregivers and calling attention to their frequently unmet needs [25]. Since this subject is less frequently discussed in Saudi Arabia, we proceeded to conduct this study aiming to assess how amputation affects psychosocial life and the quality of life in the amputees, psychosocial processes involved in adjusting to amputation and a prosthesis, and the burden of amputees on caregivers in Saudi Arabia.

## Materials and methods

Participants

Participants in this study were from Saudi Arabia who had their LLAs and caregivers who were over the age of 18. The cross-sectional study was conducted from November 2021 to February 2022, and it included all amputees and caregivers available at the time of the study. A total of 458 participants were included in the study provided they agreed to participate in the study, both Saudi and non-Saudi nationals, and were above the age of 18. The exclusion criteria included any participants below 18 years old. The Institutional Review Board at King Faisal University authorized this research with research No. KFU-REC-2021-OCT-EA00073. The research's purpose was explained to the participants, and informed permission was taken prior to the start of the research.

Measures

Amputee-Related Questionnaire

Structured questionnaires were used to gather information. They contained sociodemographic and medical data on the participants (gender, age, educational, marital, and work status, as well as any comorbidities). They also included information about amputations, such as the type of amputation, reason for amputation, time since receiving a prosthesis, if any, residual limb pain, and phantom limb pain.

Quality of Life

Short-Form Health Survey (SF-12) was used to assess QOL, and it is an internationally validated tool. Eight scales make up the multifunctional SF-12, a short health survey with two summary scores, the physical component summary (PCS) and the mental component summary scores (MCS) [26].

The Trinity Amputation and Prosthesis Experience Scales (TAPES)

A validated tool for use in individuals after amputation was used to measure the adaptations to the amputation and the prosthetic limb, TAPES is a multidimensional questionnaire that evaluates adjustment to amputation and prosthesis use. It consists of three psychosocial adjustments, three activity restrictions, and three prosthesis satisfaction subscales and was developed specifically to be used with people who have had a lower limb amputation [27].

Depression, Anxiety, Stress Scale (DASS)

This 42-item questionnaire was used by Lovibond et al. [28] in 1995 to assess levels of stress, anxiety, and depression. Its abbreviated form contains 21 questions, with seven questions assigned to each of the feelings of depression, anxiety, and stress, and is evaluated on a 4-point scale from 0 to 3.

Caregiver-Related Questionnaire

The caregiver's age, gender, level of education, monthly income, relationship to the amputees, period of care, time spent together per day, and the presence of any diagnosed physical or psychological disease, as well as the use of a sociodemographic data form.

The Zarit Burden Interview (12-item ZBI)

ZBI questionnaire was used to assess the amount of care burden in chronic diseases. The ZBI is a commonly used measure for describing caregiver burden that has been well-validated in a variety of caregivers. A score of ≥17 is suggested as an indicator of high caregiver burden [29].

Statistical analysis

Following data extraction, it was reviewed, coded, and entered into Statistical Package for Social Sciences (SPSS) version 22 (IBM Corp., Armonk, NY, USA). All statistical analysis was done using two-tailed tests. P value less than 0.05 was statistically significant. All parameters, involving respondent age, gender, insurance, nationality, living situation, psychological status, and amputation level, were subjected to descriptive analysis depending on frequency and percent distribution. Also, medical history was graphed using bar charts. A mean score with standard deviation was used to display TAPES among study patients. The frequency table for prosthesis data including the level of satisfaction, duration, and related co-morbidities was displayed in the frequency table. SF-12 was graphed for its main domains (PCS and MCS) using the box-plot diagram. Relations between TAPES, and SF-12 PCS and MCS scores were tested using Pearson correlation coefficient analysis. Mental health including the level of depression, anxiety, and stress was assessed and frequencies using the DASS scale after summing up all discrete items scores for each domain and categorizing reference to the original scale-guided cut-off points. One-way ANOVA was used to test for a significant relation between PCS and MCS subscales and patients’ mental health. To determine the relationship between DASS and SF-12, an independent t-test was performed. Crosstabulation was used to assess the distribution of amputee's psychological health status by their use of prosthetics. Relationships were examined using the exact probability test for small frequency distributions and the Pearson chi-square test.

## Results

Table 1 shows the bio-demographic data of amputees who underwent amputation in Saudi Arabia. A total of 21.8% of the study group was aged 18-25 years, 25.1% aged 36-45 years, and only 14.2% aged more than 55 years. 69.9% of amputees were males and 84.9% were Saudi. Considering marital status, 38.1% were single, and 47.3% were married. As for educational level, 18.4% had below the secondary level of education while 34.3% had a university level of education. A total of 28.5% of the amputees were retired, and 23.4% were employed while 14.6% were not working. Exactly 45.6% had insurance and 18% were diagnosed with psychological problems. A total of 79.1% live with their families and 5.9% live alone.

**Table 1:** Bio-demographic data of amputees who underwent amputation in Saudi Arabia

Bio-demographic data	No.	%
Age in years		
18-25	52	21.8%
26-35	48	20.1%
36-45	60	25.1%
46-55	45	18.8%
> 55	34	14.2%
Gender		
Male	167	69.9%
Female	72	30.1%
Nationality		
Saudi	203	84.9%
Non-Saudi	36	15.1%
Marital status		
Single	91	38.1%
Married	113	47.3%
Divorced / widow	35	14.6%
Educational level		
Below secondary	44	18.4%
Secondary	113	47.3%
University / above	82	34.3%
Job type		
Not working	35	14.6%
Student	37	15.5%
Working	56	23.4%
Undefined	43	18.0%
Retired	68	28.5%
Do you have insurance?		
Yes	109	45.6%
No	130	54.4%
Have you been diagnosed with psychological problems?		
Yes	43	18.0%
No	196	82.0%
Living situation		
With family	189	79.1%
With partner	36	15.1%
Alone	14	5.9%

Table 2 shows a total of 42.3% of the amputees had below-knee amputation, 26.4% had amputation above the knee, through the knee among 17.2%, and bilateral among 14.2% of amputees. The most reported causes of amputation were trauma (39.3%), followed by DM (23.4%), vascular diseases (12.1%), and cancer reported at 7.1%. By the time of the study, an exact 19.7% of the participants had their amputation for less than one year, 46% had amputation for one to five years, and 34.3% had amputation for over five years. A total of 43.5% of the amputees used prosthetics. A total of 24% used prostheses for less than one year, 44.2% used them for one to five years, and 13.5% used them for more than 10 years. Exactly 27.9% of the amputees who used prostheses experienced residual limb pain and 61.5% experienced phantom limb pain.

**Table 2:** Amputation data among study patients undergone amputation, Saudi Arabia. a: only amputees with prosthesis DM: diabetes mellitus

Bio-demographic data	No.	%
level of amputation		
Below knee	101	42.3%
Above knee	63	26.4%
Through knee	41	17.2%
Bilateral lower limb	34	14.2%
Cause of amputation		
Trauma	94	39.3%
DM	56	23.4%
infection	11	4.6%
Vascular disease	29	12.1%
Cancer	17	7.1%
Others	32	13.4%
How long have you had the amputation?		
< 1 year	47	19.7%
1-5 years	110	46.0%
> 5 years	82	34.3%
Do you Use of prosthetics?		
Yes	104	43.5%
No	135	56.5%
time since received a prosthesis? ^a^		
< 1 year	25	24.0%
1-5 years	46	44.2%
5-10 years	19	18.3%
> 10 years	14	13.5%
Do you experience residual limb pain? ^a^		
Yes	29	27.9%
No	75	72.1%
Do you experience phantom limb pain? ^a^		
Yes	64	61.5%
No	40	38.5%

**Figure 1:**
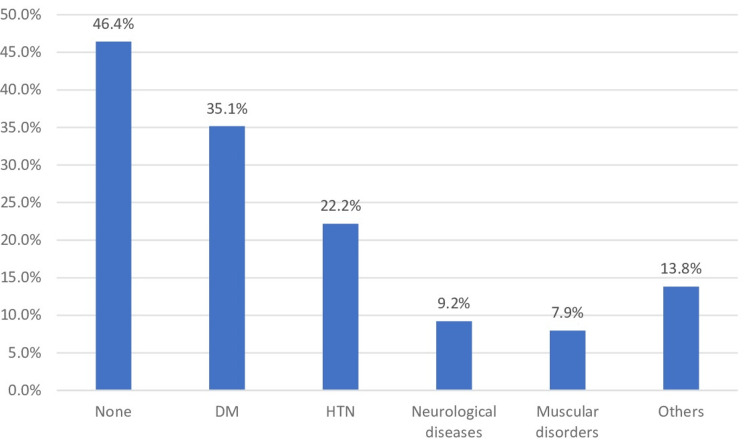
Chronic health problems among amputees undergone amputation, Saudi Arabia. DM: diabetes mellitus, HTN: hypertension.

Figure 1 shows chronic health problems among amputees undergone amputation, Saudi Arabia. The most reported chronic health problem was DM (35.1%), followed by hypertension (HTN) (22.2%), neurological diseases (9.2%), and muscular disorders (7.9%); 46.4% had no chronic health problem.

**Figure 2:**
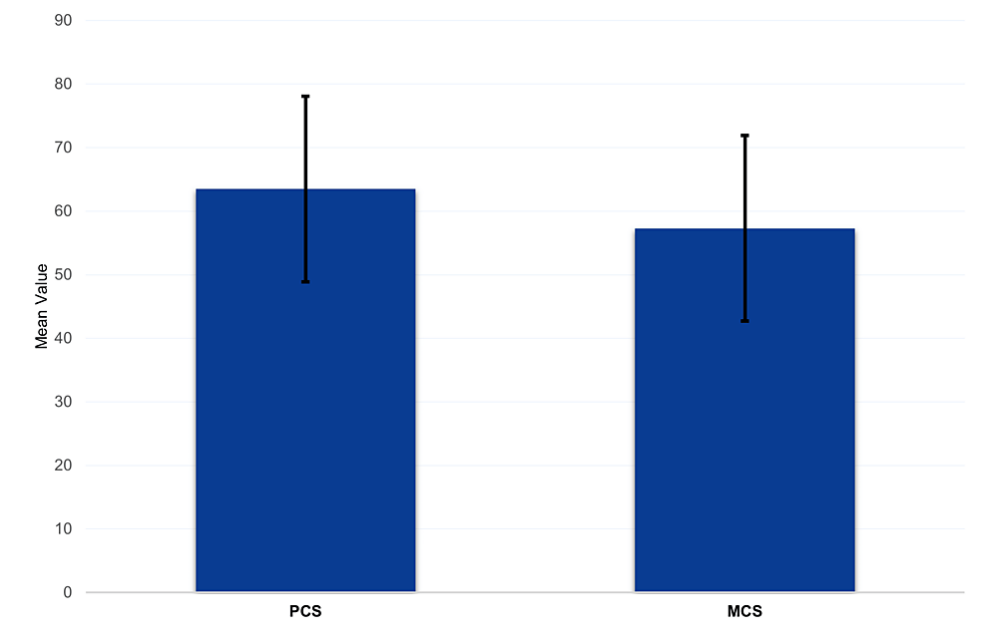
SF-12 subscales among patients undergone lower limb amputation. MCS: mental component summary, PCS: physical component summary, SF-12: Short Form-12

Figure 2 shows that the average level of the PCS was 63.5% ± 14.6% and the MCS was 57.3% ± 12.9%.

Table 3 shows correlations between TAPES, SF-12 PCS and MCS scores. The table shows that there is a significant positive correlation between psychological adjustment domains and total with PCS and MCS dimension of QOL with correlation co-efficient ranging from 0.19 to 0.48. Only adjustment to limitation were insignificantly correlated with PCS domain (r=-.088; P > 0.05). Additionally, satisfaction with prosthesis with its domains showed a significant positive correlation with PCS and MCS domains of QOL with correlation co-efficient ranging from 0.33 to 0.46. Activity restriction had a significant negative correlation with PCS (r=-0.37) and with MCS (r=-0.34).

**Table 3:** Correlations between TAPES and SF-12 PCS and MCS scores * P < 0.05 (significant)  ** P < 0.001 (highly significant) TAPES: Trinity Amputation and Prosthesis Experience Scales, SF-12: Short Form-12, MCS: mental component summary; PCS: physical component summary

TAPES	PCS	MCS
1-Psychosocial adjustment total		
1-General adjustment	.480**	.412**
2-Social adjustment	.378**	.321**
3-Adjustment to Limitation	-.088	.192*
2-Activity Restriction	-.374**	-.341**
3-Satisfaction with Prosthesis		
1-Aesthetic satisfaction	.463**	.327**
2-Functional restriction	.363**	.330**

Table 4 shows the psychological health among amputees with LLAs in Saudi Arabia. As for depression, it was mild to moderate among 42.7% of the amputees, severe among 20.5%, and 36.8% had no depression at all. A total of 31.8% of amputees had mild to moderate anxiety, 26.4% had severe anxiety but 41.8% had no anxiety. The stress level was mild to moderate among 26.8% of amputees, severe among 10.9%, and 62.3% had no stress.

**Table 4:** Psychological health among amputees with lower limb amputation, Saudi Arabia.

Psychological health	No.	%
Depression severity		
Normal	88	36.8%
Mild / moderate	102	42.7%
Severe / extremely severe	49	20.5%
Anxiety level		
Normal	100	41.8%
Mild / moderate	76	31.8%
Severe / extremely severe	63	26.4%
Stress level		
Normal	149	62.3%
Mild / moderate	64	26.8%
Severe / extremely severe	26	10.9%

Table 5 shows the distribution of QOL subscales by psychological health among amputees who had undergone lower limb amputation. Depression showed a significant association with the PCS domain where the highest mean score of PCS was among those with no depression (67.3 ± 16.7) versus 58.4 ± 12.1 for others with severe depression (P=.002). Anxiety was significantly associated with both PCS and MCS domains of QOL. The mean PCS score was 67.7 ± 17.3 among patients without anxiety compared to 61.6 ± 12.2 for others with severe anxiety (P=.001). Also, the mean score of MCS among patients with no anxiety was 59.4 ± 14.7 in comparison to 57.3 ± 11.4 among others with severe anxiety (P=.038). Stress had no significant relationship with any of the QOL domains.

**Table 5:** Distribution of QOL subscales by psychological health among amputees who had undergone lower limb amputation. P: One Way ANOVA  * P < 0.05 (significant) MCS: mental component summary, PCS: physical component summary, QOL: quality of life, DASS-21: Depression, Anxiety, Stress Scale

DASS-21	PCS		MCS	
	Mean	SD	Mean	SD
Depression severity				
Normal	67.3	16.7	56.7	15.6
Mild / moderate	62.7	12.9	58.2	10.1
Severe / extremely severe	58.4	12.1	56.2	12.6
p-value	.002*		.578	
Anxiety level				
Normal	67.7	17.3	59.4	14.7
Mild / moderate	59.5	10.8	54.4	10.9
Severe / extremely severe	61.6	12.2	57.3	11.4
p-value	.001*		.038*	
Stress level				
Normal	64.7	15.4	56.8	14.0
Mild / moderate	60.8	11.8	57.6	9.8
Severe / extremely severe	63.5	15.9	58.8	12.9
p-value	.207		.740	

Table 6 shows the distribution of amputees' psychological health status by their use of prosthetics. Only stress showed a significant relation with prosthetics use as 21.2% of the prosthetics users experienced mild/moderate stress compared to 31.1% of non-users and 17.3% of the users had severe stress versus 5.9% of non-users with recorded statistical significance (P=.010). Depression and anxiety showed a non-significant association with prosthetics used.

**Table 6:** Distribution of amputee's psychological health status by their use of prosthetics. P: Pearson X2 test  * P < 0.05 (significant)

Psychological health	Do you Use of prosthetics?				p-value
	Yes		No		
	NO.	%	NO.	%	
Depression severity					.336
Normal	33	31.7%	55	40.7%	
Mild / moderate	49	47.1%	53	39.3%	
Severe / extremely severe	22	21.2%	27	20.0%	
Anxiety level					.383
Normal	40	38.5%	60	44.4%	
Mild / moderate	32	30.8%	44	32.6%	
Severe / extremely severe	32	30.8%	31	23.0%	
Stress level					.010*
Normal	64	61.5%	85	63.0%	
Mild / moderate	22	21.2%	42	31.1%	
Severe / extremely severe	18	17.3%	8	5.9%	

Table 7 shows the relation between prosthetics uses among amputees with their psychological health and QOL. Psychological health showed insignificant association with prosthetics use neither for depression, stress not anxiety. Considering the quality of life, PCS mean score was significantly higher among prosthetics users than among non-users (68.2 ± 15.5 vs. 59.9 ± 12.8, respectively; P=.001). Also, the MCS score was significantly higher among prosthetics users than among non-users (59.5 ± 12.4 vs. 55.5 ± 13.0, respectively; P=.001).

**Table 7:** Relation between prosthetics use among amputees with their psychological health and quality of life. P: Independent t-test  * P < 0.05 (significant) MCS: mental component summary, PCS: physical component summary, DASS-21: Depression, Anxiety, Stress Scale, SF-12: Short Form-12

DASS & SF-12	Do you use prosthetics?				p-value
	Yes		No		
	Mean	SD	Mean	SD	
Psychological health					
Depression	7.0	4.9	6.3	4.9	.315
Anxiety	5.7	4.7	4.9	4.3	.176
Stress	6.9	5.2	6.3	4.4	.365
Quality of life					
PCS	68.2	15.5	59.9	12.8	.001*
MCS	59.5	12.4	55.5	13.0	.018*

Table 8 shows the bio-demographic data of amputee caregivers, Saudi Arabia. A total of 34.7% of the caregivers were aged 18-25 years, 32% aged 26-35 years and only 3.2% were aged above 55 years. As for marital status, 49.8% were single and 42.9% were married. Considering education level, 6.8% had below the secondary level of education, 29.7% had secondary level while 63.5% were university graduates. About caregivers’ work, 24.2% were not working/retired while 32% were students and 43.8% were working. A total of 37.9% of caregivers had medical insurance. The most reported chronic diseases among caregivers were DM (10.5%) and HTN (6.8%), while 76.3% had no chronic health problems. An exact 11.4% of the caregivers were diagnosed with psychological disease.

**Table 8:** Bio-demographic data of caregivers, Saudi Arabia

Bio-demographic data	No	%
Age in years		
18-25	76	34.7%
26-35	70	32.0%
36-45	47	21.5%
46-55	19	8.7%
> 55	7	3.2%
Gender		
Male	105	47.9%
Female	114	52.1%
Nationality		
Saudi	198	90.4%
Non-Saudi	21	9.6%
Marital status		
Single	109	49.8%
Married	94	42.9%
Divorced / widow	16	7.3%
Education level		
Below secondary	15	6.8%
Secondary	65	29.7%
University / above	139	63.5%
job		
Not working / retired	53	24.2%
Student	70	32.0%
Working	96	43.8%
Monthly income		
< 5000 SR	67	30.6%
5000-10000 SR	74	33.8%
10000-20000 SR	51	23.3%
> 20000 SR	27	12.3%
Had medical insurance?		
Yes	83	37.9%
No	136	62.1%
Have chronic diseases?		
None	167	76.3%
DM	23	10.5%
HTN	15	6.8%
Musculoskeletal disorders	2	.9%
Neurological disorders	6	2.7%
Others	23	10.5%
Had ever diagnosed with psychological disease?		
Yes	25	11.4%
No	194	88.6%

Table 9 shows data reported by caregivers on caring for amputees in Saudi Arabia. A total of 20.1% were amputee's son/daughter, 14.6% were their brother/sister, 13.2% were the couple while 52.1% of the caregivers were others. 54.3% of caregivers cared for amputees who had below-knee amputation, 13.7% for through-the-knee amputees, and 14.6% for bilateral amputees. The most reported cause of amputation was DM (45.2%), accidents (25.1%), and vascular diseases (8.7%). Exactly 37.4% of the caregivers served the amputee for less than one year, 24.7% for one to two years, while 19.6% cared for the amputee for five years or more. Care started after amputation among 58.4% of caregivers. Daily sleep hours for less than six hours were reported by 58.9% of caregivers and 74% of caregivers got help in caring for amputees. A total of 29.2% of caregivers practice sports once per week, 21.9% for two to three times/week, and 12.8% practice daily while 36.1% never practice sports. As for daily spent hours spent caring for amputees, it was less than one hour among 25.6% of caregivers, one to two hours among 33.8%, three to four hours among 20.1%, and more than four hours among 20.5%.

**Table 9:** Data reported by caregivers on caring for amputees in Saudi Arabia DM: diabetes mellitus

Amputee carrying data	No	%
Relationship with the amputee		
Husband / wife	29	13.2%
Son / daughter	44	20.1%
Brother / sister	32	14.6%
Others	114	52.1%
Type of amputation		
Below knee	119	54.3%
Through knee	30	13.7%
Above knee	38	17.4%
Bilateral lower limbs	32	14.6%
Cause of amputation		
Accident	55	25.1%
DM	99	45.2%
Vascular disease	19	8.7%
Tumor	12	5.5%
Infection	6	2.7%
Others	28	12.8%
Amputee uses prosthetics		
Yes	78	35.6%
No	141	64.4%
Duration of caring for the amputee		
< 1 year	82	37.4%
1-2 years	54	24.7%
3-4 years	40	18.3%
5 or more years	43	19.6%
When did you start caring for amputee		
Before amputation	91	41.6%
After amputation	128	58.4%
Daily sleep hours		
< 6 hours	129	58.9%
> 6 hours	90	41.1%
Do you get help in caring for amputee?		
Yes	162	74.0%
No	57	26.0%
Frequency of practicing sports / week		
Never / infrequent	79	36.1%
Once / week	64	29.2%
2-3 times / week	48	21.9%
Almost daily	28	12.8%
Daily hours spent caring for amputee		
< 1 hour	56	25.6%
1-2 hours	74	33.8%
3-4 hours	44	20.1%
> 4 hours	45	20.5%

**Figure 3:**
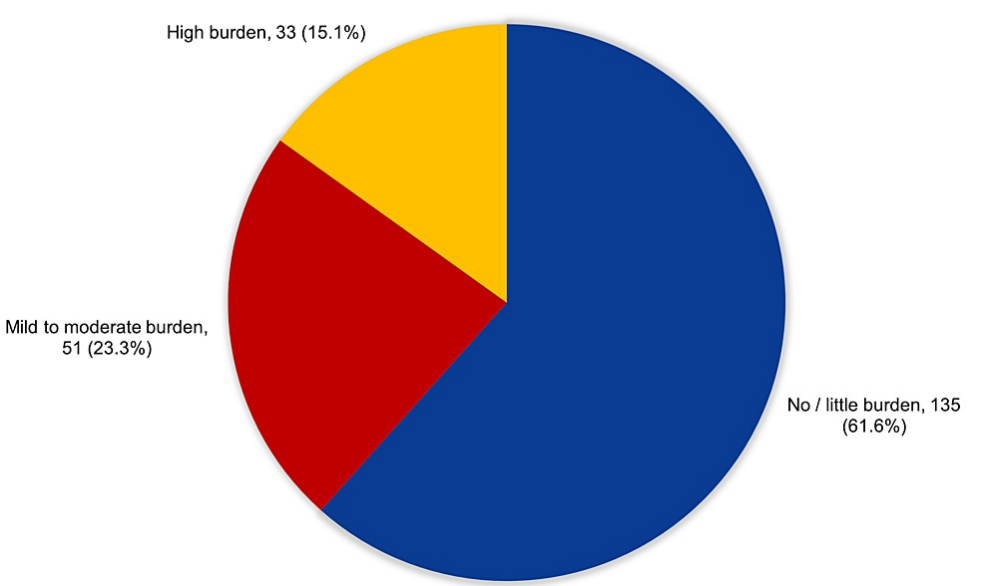
Overall degree of burden among amputee caregivers, Saudi Arabia.

Figure 3 shows a total of 15.1% of caregivers experienced high burden, while 23.3% had mild to moderate burden, but 61.6% had no or little burden.

## Discussion

QOL and TAPES

Individuals who underwent lower limb amputations exhibited worse QOL when compared to the entire population. This result has been supported by several additional research, suggesting that amputation is a major life event that can have long-term effects on QOL [30-33]. Amputation of a limb is a life-altering event having social, mental, psychological, and spiritual consequences [1]. These problems originate from their incapacity to carry out everyday tasks, remain independent, and support their families [34]. According to a large cohort research done in Saudi Arabia, the country is seeing a significant growth in type II diabetes, with a prevalence placing among the top 10 countries worldwide. Furthermore, the prevalence of diabetes-related morbidities is among the highest in the world, with 2.05% for foot ulcers and 1.06% for amputations. When compared to individuals with diabetic foot ulcers, amputees had the lowest overall survival rate [35-36]. In the QOL, the average level of the PCS was higher than the MCS in this study. In our study, there is a significant positive association between psychological adjustment dimensions and satisfaction level with prosthesis with PCS and MCS dimensions QOL. While there is a significant negative correlation between activity restriction with PCS and MCS. In a similar study by Sinha et al. [37] the effects of social adjustment, adjustment to limitation, and functional restriction on QOL were shown to be significant. Both PCS and MCS are significantly and positively linked with functional restriction, while PCS is more strongly associated. Social adjustment is linked to both PCS and MCS in a significant and positive direction, with MCS having a greater correlation. Gallagher and MacLachlan (2004) reported that functional restriction was significantly and negatively linked with the physical health domain of The World Health Organization Quality of Life (WHO-QOL) [38]. Functional restriction has been shown to be connected significantly and negatively with the physical, psychological, and social health areas of WHO-QOL in research by Deans et al. [39]. The physical aspect of QOL was additionally found to be more negatively affected by the activity restriction scale. Moreover, Weiss et al. [40] discovered that the capacity to do daily activities is the most significant indicator of QOL. Deans et al. [39] found that restricting athletic activity had a significant detrimental impact on the physical health area of WHO-QOL. Activity restriction and aesthetic satisfaction were shown to have the greatest impact on QOL, whereas social adjustment and, to a lesser extent, functional restriction influenced QOL. PCS and MCS of QOL may be affected by residual stump pain and phantom limb pain. Residual limb pain and phantom limb pain can be postulated to affect the physical and mental components of QOL. In our study,27.9% of the participants who use prostheses experienced residual stump pain, and 61.5% of the participants who use prostheses experienced phantom limb pain. Phantom pain becomes less common as time passes after amputation [31]. When it came to the QOL, prosthesis users had a considerably higher PCS mean score than non-users. In addition, prosthesis users had a significantly higher MCS score than non-users.

The Depression, Anxiety and Stress Scale - 21 Items (DASS-21) 

A study discovered a strong association between unsuccessful prosthetic fitting and a number of psychological problems, such as depression [41]. Our study found that 20.5% of participants had severe to extremely severe depression, whereas 42.7% had mild to moderate depression. In this study, 41.8% of respondents reported normal anxiety, 31.8% showed mild to moderate anxiety, and 26.4% revealed severe to extreme anxiety. According to a previous study, 35.5% of participants had significant anxiety [42]. Normal stress was recorded by 62.3% of participants, mild to moderate stress was observed by 26.8%, and severe to extreme stress was reported by 10.9%. The findings revealed that people without prostheses had greater levels of severe depression. whereas those who use prostheses have greater levels of severe anxiety and stress. If we compare based on QOL, it will show that participants with mild to moderate and severe to extremely severe depression have better PCS values than MCS. Also, PCS is higher than MCS in participants with mild to moderate and severe to extremely severe anxiety and stress. Horgan and Maclachlan [1] found in their review of psychological adjustments to amputation that depression and anxiety seem to be relatively high up to two years following amputation, they drop thereafter to levels equivalent to those in the general community. Additionally, there was a substantial difference in the prevalence of depression and anxiety, which peaked after amputation and then decreased throughout inpatient rehabilitation before rising once again after release [21].

Caregivers

According to the findings, more than half of the participants (61.6%) experienced no or minimal burden. The main reason may be because most of the caregivers in our study are below age 35. Only 15.1% of participants had a high burden, while 23.3% had a mild to moderate burden. Regarding the duration of carrying the amputees, our study shows that 37.4% of the caregivers served the amputee for less than one year, 24.7% for one to two years, while 19.6% cared for the amputee for five years or more. Ojoawo et al. [43] studied and analyzed the caregiving load between unofficial carers of people with varying degrees of amputation. They discovered that females had a somewhat higher-than-average but considerably greater score than men. Also, they noticed the caregiver burden was the greatest in cases of above the knee amputations and the smallest in cases of below-knee amputations. In terms of daily hours spent carrying an amputee, 25.6% of carers spent less than one hour, 33.8 percent spent one to two hours, 20.1% spent three or four hours, and 20.5% spent more than four hours. Mainly because most of the caregiver are students and others are working and they have less time to care them, and they are busy with their personal life. Longer care is linked to greater caregiver burden and depression [44]. This study shows that 45.2% of caregivers care the amputees and the primary cause is DM. In this research, 58.9% of the participants had a daily sleep duration of fewer than six hours. According to a different study, 50% of the carers slept for up to six hours per night [45]. In this study, 36.1% of caregivers never play sports, compared to 29.2% who play once per week, 21.9 % who play two to three times, and 12.8% who play every day. This might be linked to the time spent in caregiving, where there are no specific hours of care, therefore it may reduce their free time and their ability to exercise and play sports. Physical activity improves patients' quality of life by having a beneficial impact on their physical ability and caregiver burden [46]. Caregivers who engaged in physical exercise at least once per week had a greater mental QOL with regard to the method of adjustment and adaptation to the disease. As it encourages breaks from the crowded and stressful daily schedules and fosters interaction, physical activity does really benefit the mental health of those who engage in it [47-49].

The strength of this study is it's being one of the first studies carried out in Saudi Arabia to assess the psychosocial life and the quality of life in amputees, the psychosocial processes involved in adjusting to amputation and a prosthesis, and the burden of amputees on caregivers. The limitations of this study are that the response rate was not good from the amputees and caregivers. Also, there was a lack of research about amputees and caregivers conducted in Saudi Arabia, and no studies were conducted to evaluate QOL in other causes of amputation because there are few studies about ulcers and amputation that related to diabetic foot.

## Conclusions

In clinical practice, it is highly advised that prosthetic limbs be evaluated using TAPES to evaluate how well they are being adjusted to and appreciated. It shows how much the quality of life for those who have had amputations may be enhanced. Our finding shows there are correlations between TAPES and SF-12 PCS and MCS scores. It is advised that psychological assessment and counseling should be a component of the overall care of amputees due to mild to moderate rates of anxiety, depression, and stress among amputees. The findings emphasize the necessity of psychological and social support to be considered in caregivers’ health assessments. Also, the physical health of the caregivers should be fundamental in their lives as it minifies the caregiver burden. Further studies should be considered with a larger sample of amputees and longitudinal studies to evaluate the adaptation changes over time, caregiving burden, and family functioning.
